# Genistein Restricts the Epithelial Mesenchymal Transformation (EMT) and Stemness of Hepatocellular Carcinoma via Upregulating miR-1275 to Inhibit the EIF5A2/PI3K/Akt Pathway

**DOI:** 10.3390/biology11101383

**Published:** 2022-09-22

**Authors:** Xiao Yang, Wangjie Jiang, Xiangxu Kong, Xiao Zhou, Deming Zhu, Lianbao Kong

**Affiliations:** 1 Hepatobiliary Center, The First Affiliated Hospital of Nanjing Medical University, Nanjing 210000, China; 2 Key Laboratory of Liver Transplantation, Chinese Academy of Medical Sciences, NHC Key Laboratory of Living Donor Liver Transplantation, Nanjing Medical University, Nanjing 210000, China

**Keywords:** hepatocellular carcinoma, miR-1275, epithelial mesenchymal transformation, stemness, EIF5A2

## Abstract

**Simple Summary:**

Genistein is a natural phytoestrogen with various antitumor effects. Our study focused on exploring the mechanisms of microRNAs and genistein to inhibit the epithelial mesenchymal transformation (EMT) and stemness of hepatocellular carcinoma (HCC). We found that miR-1275 was more highly expressed in HCC cells treated with genistein compared with the control. Then, we performed series functional experiments to explore the relationship between genistein and miR-1275 in HCC. The inhibition of genistein on HCC cells was enhanced by the increase in treatment time and dose, and miR-1275 can be raised by genistein. The overall survival and recurrence-free survival of HCC patients with low expressed miR-1275 were lower than those of those with high expression levels. The experimental results exhibited that genistein and miR-1275 can both significantly suppress the proliferation, migration, invasion, metastasis, EMT and stemness of HCC. Moreover, the inhibition can be further enhanced with the co-existence of miR-1275 mimic and genistein. Finally, we demonstrated that miR-1275 can inhibit the EMT and stemness of HCC via inhibiting the EIF5A2/PI3K/Akt pathway. Our findings proved that genistein can inhibit the EIF5A2/PI3K/Akt pathway by upregulating miR-1275 so as to attenuate the EMT and stemness of HCC cells to restrict their progression and metastasis.

**Abstract:**

**Purpose:** Genistein is a natural phytoestrogen with various antitumor effects. In recent years, some microRNAs (miRNA) in cancer cells have been reported to be regulated by genistein. Our study focused on exploring the mechanisms of miRNA upregulation to inhibit the epithelial mesenchymal transformation (EMT) and stemness of hepatocellular carcinoma (HCC). **Patients and Methods:** MiR-1275 was discovered by the transcriptome sequencing of miRNA expression profiles in HepG2 cells treated with genistein or DMSO as a control. Then, we performed series functional experiments in vitro and vivo to explore the relationship between genistein and miR-1275 in HCC. The target gene (Eukaryotic initiation factor 5A2, EIF5A2) of miR-1275 was predicted by databases and finally determined by a dual luciferase reporter assay. The downstream signaling pathway of EIF5A2 was assessed by bioinformatics analysis and Western blot. **Results:** the inhibition of genistein on the viability of HCC cells was enhanced by the increase in treatment time and dose, but it had no obvious inhibitory effect on normal hepatocytes (QSG-7701). Through qRT-PCR and transcriptome sequencing, we discovered that miR-1275 was lowly expressed in HCC, and it can be raised by genistein. The overall survival (OS) and recurrence-free survival (RFS) of HCC patients with lowly expressed miR-1275 were lower than those of those with high expression levels. In vitro and vivo experiments exhibited that genistein and the overexpression of miR-1275 can both significantly suppress the proliferation, migration, invasion, metastasis, EMT and stemness of HCC. Moreover, the inhibition can be further enhanced when miR-1275 mimic and genistein exist together. Finally, we demonstrated that miR-1275 can inhibit the epithelial mesenchymal transformation (EMT) and stemness of HCC via inhibiting the EIF5A2/PI3K/Akt pathway. **Conclusion:** Our findings proved that genistein can inhibit the EIF5A2/PI3K/Akt pathway by upregulating miR-1275 so as to attenuate the EMT and stemness of HCC cells to restrict their progression and metastasis.

## 1. Introduction

Hepatocellular carcinoma (HCC) ranks second among all cancers that can cause cancer-related death [1]. The poor prognosis of HCC is attributed to multiple pathogenic factors, the high recurrence and the metastasis rate [2]. Although great progress has been made in the research of surgical strategies and targeted therapeutic drugs, the coping strategies for the recurrence and metastasis of HCC are still limited [3]. Epithelial mesenchymal transformation (EMT) and stemness are the key factors leading to a high recurrence and metastasis rate of HCC. EMT refers to the process of transforming tumor cells with epithelial characteristics into cells with mesenchymal characteristics. In this process, tumor cells lose the polarity and the ability of adhesion to the basement membrane and acquire stronger characteristics of mobility and metastasis [4]. The stemness of tumor cells is defined as the ability of self-renewal, multi-lineage differentiation potential and tumor initiation, which is essential for tumor recurrence and metastasis. Furthermore, the stemness is plastic and can be affected by diversified factors, such as hypoxia and EMT [3,5,6]. It has been reported that the activation of classical signaling pathways responsible for EMT such as Akt, TGF-β, C-MYC and Notch can also improve the stemness of tumor cells [7]. The enhancement of EMT and stemness usually leads to drug resistance and worse clinical outcomes, so it is imperative to find novel adjuvant drugs for the treatment of HCC.

Genistein, a natural compound extracted from soybean, is the member of the soybean isoflavone family with the most extensive biological activities and antitumor effects [8]. Previous reports have shown that genistein has the functions of protecting the cardiovascular system, regulating blood glucose, delaying neurological decline and antitumor [9,10,11,12]. In addition, recent studies on genistein have confirmed that it possesses the ability to inhibit the proliferation, invasion and metastasis of a variety of cancers, including HCC, renal cancer and breast cancer [8,13,14]. At the same time, several studies have shown that genistein can enhance the efficacy of sorafenib and reverse the resistance of HCC to sorafenib [13,15]. These reports also indicated that genistein has potential value as a new adjuvant therapy for HCC. However, the specific mechanism of genistein inhibiting tumor progression and metastasis has not been fully elucidated, especially in HCC. In our study, we speculated whether genistein can prevent the progression and metastasis of HCC by inhibiting the EMT and stemness. MicroRNAs (miRNAs) also play an important role in the regulation of the EMT and stemness of HCC, so we focused our research on the relationship between genistein and miRNAs in HCC.

MiRNAs are a group of small single stranded noncoding RNAs that are 20–24 bp in length and silence the translation products of the targeted mRNAs to adjust cell life activities. MiRNAs participate in the post transcriptional regulation of target genes, mainly by binding to the 3′UTR region of target mRNAs [16,17]. In these studies of HCC, many miRNAs have been proven to be involved in the regulation of tumor proliferation, invasion and metastasis [16,17,18]. For example, Qiongying Hu et al. [19] confirmed that miR-101 can suppress HCC progression by decreasing the expression level of VEGF. MiRNAs may become novel breakthroughs or therapeutic targets for seeking new therapeutic methods for HCC. Previous studies mainly focused on the direct effects of genistein on HCC. However, whether genistein can restrain the progression and metastasis of HCC via regulating miRNAs has not been determined. Therefore, in this study, we screened the upregulated miRNAs in HCC cells treated with genistein by sequencing and further explored whether these miRNAs play an antitumor role by inhibiting EMT and stemness. Our results revealed a new pathway for genistein to inhibit HCC progression and metastasis and also provide a theoretical basis for exploring new treatments for HCC.

## 2. Materials and Methods

### 2.1. Patients and Samples

Seventy pairs of HCC samples and their paracancerous tissues were obtained from the Department of Hepatobiliary Center, the First Affiliated Hospital of Nanjing Medical University. All HCC patients underwent surgery and were diagnosed by pathology. This study was authorized in advance by the Department of Institutional Review Board (IRB), the First Affiliated Hospital of Nanjing Medical University, and all patients and their guardians signed Informed consent forms.

### 2.2. Cell Lines and Culture

Normal hepatocytes (QSG-7701), HCC cell lines (HCC-LM3, MHCC-97H, Hep-3B, Hep-G2 and Huh-7) and HEK293T cells were acquired from the Institute of Cell Biology, Chinese Academy of Sciences (Shanghai, China). All the above cell lines were cultured in a DMEM medium (Gibco BRL, Gaithersburg, MD, USA) supplemented with high glucose, 10% fetal bovine serum (FBS) (TransGen Biotech, Beijing, China) and 1% Penicillin-Streptomycin (TransGen Biotech, Beijing, China). All these cells were cultured in a cell incubator containing 5% CO_2_ at 37 °C before experiments.

### 2.3. Genistein Treatment

Genistein (Sigma-Aldrich, St. Louis, MO, USA) was dissolved in dimethylsulfoxide (DMSO) before treating cells, and the volume fraction of DMSO was less than 0.1% in the culture medium. According to the results of cell viability experiments, the HCC cells were treated with genistein (10.81 µg/mL) or DMSO (the control group) 3 days in advance of the experiments.

### 2.4. Mimic and Plasmid Transfection

Inhibitor, inhibitor-NC, Mimic and Mimic-NC of miR-1275 (RiboBio, Guangzhou, China) were transfected into HCC cells by Lipofectamine 3000 (Invitrogen, Waltham, MA USA). After 3 days, these HCC cells were used for subsequent functional experiments. In rescue experiments, the overexpression plasmid of Eukaryotic initiation factor 5A2 (EIF5A2) (CoreusBiotech, Nanjing, China) was also transfected into HCC cells by Lipofectamine 3000. The scramble plasmid of EIF5A2 (EIF5A2-NC) (CoreusBiotech, Nanjing, China) was used as the control. These cells transfected with mimics or plasmids were prepared for subsequent in vitro experiments.

### 2.5. Lentivirus Construction and Infection

The lentivirus vectors encoding homo ov-miR-1275 (CoreusBiotech, Nanjing, China) were the constructed overexpression miR-1275, and the empty vectors (ov-vector) were used as the control group. Then, the HCC cells were transfected with lentivirus for 72 h. Then, the transfection efficiency was observed by an immunofluorescence microscope. Finally, the HCC cells were screened with puromycin (5 µg/mL) for 3 weeks until stable cell clones were established. These cells transfected with lentivirus were used for subsequent in vivo experiments.

### 2.6. Cell Viability Assay

HCC cells and normal hepatocytes (QSG-7701) were seeded into 96-well cell culture plates (3000 cells/well) and then separately treated with genistein at the concentrations of 0, 0.1, 0.5, 1, 5, 10, 50 and 100 µg/mL for 3 days. The medium was replaced with a fresh culture medium containing 10% CCK-8 solution (Vazyme, Nanjing, China) at 24 h, 48 h and 72 h, respectively, and incubated with cells at 37 °C for 2 h. The absorbance of each well was measured at 450 nm by an automatic microplate reader (ELX808, BioTek, Norcross, GA, USA) for further half maximal inhibitory concentration (IC_50_) analysis.

### 2.7. miRNAs Transcriptome Sequencing

According to the results of the cell viability test, the miRNA expression profile in Hep-G2 cells treated with genistein (10.81 µg/mL) or DMSO (control) for 3 days was analyzed by high throughput transcriptome sequencing (LC-Bio, Hangzhou, China). Three independent samples were prepared for each group in advance.

### 2.8. RNA Isolation and Quantitative Real-Time Polymerase Chain Reaction (qRT-PCR)

The total RNA isolated from samples was performed following the instructions of the RNA Extraction Kit (Vazyme, Nanjing, China). The reverse transcription synthesis of cDNA was operated under the guidance of the HiScript II Q RT SuperMix kit (Vazyme, Nanjing, China). The expression levels of miR-1275 and EIF5A2 were measured by qRT-PCR, and this assay was conducted by the 7900HT Fast Real-Time PCR System (ABI, Waltham, MA, USA) and ChamQ Universal SYBR qPCR Master Mix (Vazyme, Nanjing, China). U6 was used as the internal reference of miR-1275, and GAPDH was selected to normalize EIF5A2. The sequences of all primers (RiboBio, Guangzhou, China) are provided in the Supplementary Materials (Table S1).

### 2.9. Cell Counting Kit-8 (CCK-8) Test

The HCC cells in each group were seeded into 96-well culture plates (2000 cells/well) and cultured for 4 days. These cells were supplemented with a fresh culture medium containing 10% CCK-8 solution (Vazyme, Nanjing, China) at 0 h, 24 h, 48 h, 72 h and 96 h, respectively, and incubated at 37 °C for 2 h. Then, the absorbance of each well was detected at 450 nm by an automatic microplate reader (ELX808, BioTek, Norcross, GA, USA).

### 2.10. 5-Ethynyl-2′-deoxyuridine (EdU) Test

The HCC cells of each group in 96-well culture plates (10^4^/well) were labeled with EDU and incubated at 37 °C for 2 h. Then, these cells were fixed with 5% paraformaldehyde and stained according to the steps in the instructions of the BeyoClick^TM^ EDU-488 Cell Proliferation Kit (Beyotime, Shanghai, China). Finally, these cells were stained with DAPI for 30 min and supplemented with fresh phosphate buffered saline (PBS) (Gibco BRL, Gaithersburg, MD, USA). The number of HCC cells stained with EDU was observed under the fluorescence microscope and calculated by Image J software.

### 2.11. Scratch-Healing Test

The HCC cells in each group were added into 6-well culture plates (Corning, Corning, NY, USA). When the density of HCC cells exceeded 95%, the tip of a 200 μL pipette gun was applied to make a scratch in the center of each well. The healing stages of scratches at 0 h, 24 h, 48 h and 72 h were observed and photographed under an inverted microscope (ZEISS, Oberkochen, Germany), and the proportion of the healing area was measured by Image J software v1.8.0 (National Institutes of Health, Bethesda, MD, USA).

### 2.12. Migration Test

The HCC cells (2 × 10^4^) and 250 uL serum-free medium were added into the upper part of each transwell chamber (8 um pore size; Corning, Corning, CA, USA). At the same time, 600 uL medium with 20% FBS was supplemented into the lower part of each chamber. After 24 h, these chambers were fixed with 5% paraformaldehyde for 30 min and then stained with crystal violet solution for 30 min. After these chambers were washed, the excess cells in the upper part of the chambers were wiped off with a cotton swab, and the migrated cells were photographed under an upright microscope. Finally, the number of migrated cells in each chamber was measured by Image J software.

### 2.13. Spheroid Formation Test

This test was performed in 24-well ultra-low adsorption culture plates with a U-shaped bottom (Corning, USA) and applied to evaluate the stemness of HCC cells. Before this test, the specific culture medium was prepared with insulin (4 ng/mL), B27, EGF (80 ng/mL), bFGF (10 ng/mL) and DMEM/F12 medium (Gibco, Waltham, MA, USA) [20]. Then, 500 μL special medium containing HCC cells (1000) was added to each well, and the medium was replaced every 2 days. After 1 week, the number of spheroids in each well was photographed and counted by an inverted microscope (ZEISS, Germany).

### 2.14. 3D Spheroid Invasion Test

This test was conducted to assess the invasive ability of HCC cells and operated in 96-well culture plates (Corning, USA). The HCC cell spheroids were centrifuged and resuspended in DMEM medium for use, and the pH value of type I collagen (Corning, USA) was adjusted to 7–7.5. Then, the HCC cell spheroid suspension and type I collagen solution were mixed in equal proportion and added to 96-well culture plates. Then, 150 μL DMEM culture medium was added into each well after the plates were incubated at 37 °C for 30 min. After 2 days, the invasion area of each spheroid was observed by an inverted microscope (ZEISS, Germany) and counted by Image J software.

### 2.15. Comet Test

This assay was performed to evaluate the effect of genistein and miR-1275 on the DNA damage and apoptosis of HCC cells. The Comet Assay was conducted on the basis of the instructions of the Comet Assay kit (KeyGEN BioTECH, Nanjing, China). The HCC cells immobilized on glass slides containing three layers of agarose gel were electrophoresed in an alkaline solution for 20 min and washed three times with a neutralizing solution. Then, these cells were stained with DAPI for 30 min and photographed under a fluorescent microscope (Leica, Wilmington, NC, USA). The Olive tail moment (the degree of DNA damage) was measured by CASP software.

### 2.16. Flow Cytometry Analysis

This assay was performed to explore the effects of genistein and miR-1275 on the cell cycle and proliferation of HCC cells. The HCC cells (10^5^/well) in each group were seeded into 6-well culture plates (Corning, USA) and treated with genistein (10.81 µg/mL) or transfected with miR-1275 mimic. After 3 days, these HCC cells were trypsinized, fixed with absolute ethanol and stored at −20 °C. Then, HCC cells were washed and stained with PropidiumIodide (PI) following the guidance of the instructions of the Cell Cycle Staining Kit (MULTISCIENCES, Hangzhou, China). The distribution of the HCC cell cycle was counted by the FACSCanto^TM^ II flow cytometer (BD, Franklin Lakes, NJ, USA), and the results were analyzed by ModFit LT software 3.1 (Verity Software House, Mountain View, CA, USA).

### 2.17. Western Blot (WB) Analysis

The primary antibodies involved in the Western blot (WB) are as follows: anti-EIF5A2 (1:2000), anti-PI3K (1:1000), anti-Akt (1:1000), anti-p-Akt (1:1000), anti-SOX2 (1:1000), anti-BMI1 (1:1000) and anti-OCT4 (1:1000) were used (Abcam, Cambridge, UK); anti-E-cadherin (E-cad) (1:1000), anti-N-cadherin (N-cad) (1:1000), anti-Vimentin (Vim) (1:1000) and anti-β-actin were used (Proteintech, Wuhan, China); the secondary antibody was HRP-IgG (1:3000) (Proteintech, Wuhan, China).

### 2.18. Xenografts of HCC In Vivo

There were 20 6-week-old male BALB/c nude mice purchased from the Animal Experiment Center of Nanjing Medical University (Nanjing, China), and they were randomly assigned to 4 groups (ov-vector, ov-miR-1275, ov-vector + Genistein and ov-miR-1275 + Genistein, *n* = 5). These nude mice were subcutaneously injected with HCC-LM3 cells (10^6^ cells/100μL, 100 μL/mouse). Before injection, HCC-LM3 cells were transfected with the lentivirus of ov-vector or ov-miR-1275 in advance. In the genistein treatment group, each nude mouse was intraperitoneally injected with 100 μL genistein (10.81 µg/mL) solution every 3 days. After 35 days, these subcutaneous HCC tumors obtained from the sacrificed mice were fixed with formaldehyde and embedded in paraffin. These specimens were sectioned and stained with haematoxylin & eosin (H&E) and immunohistochemistry (IHC) for EIF5A2, Ki-67, PI3K, p-Akt, SOX2 and Vimentin. This animal experiment was approved by the Animal Ethics Committee of Nanjing Medical University (Nanjing, China).

### 2.19. Lung Metastasis Model of HCC In Vivo

As described above, a total of 20 6-week-old male BALB/c nude mice were randomly divided into 4 groups. These nude mice were injected with HCC-LM3 cells via the caudal vein, and the administration method of genistein was the same as that described above. After 50 days, these mice were euthanized, and their lung tissues were removed and photographed. These lung tissues were sectioned and stained with H&E. The numbers of metastatic nodules in lung tissues were counted under an orthographic microscope. This animal experiment was also approved by the Animal Ethics Committee of Nanjing Medical University (Nanjing, China).

### 2.20. Dual Luciferase Reporter Test

The plasmids (labeled with firefly luciferase) containing wild-type or mutant fragments of EIF5A2 3′-UTR and miR-1275 mimic or NC duplex (labeled with Renilla luciferase) were co-transfected to HEK293T cells for 2 days. Then HEK293T cells were lysed, and the luciferase activity was measured under the guidance of the instructions of the Bio-Lite Luciferase Assay System (Vazyme, Nanjing, China) by a fluorometer.

### 2.21. Online Public Database and Bioinformatics Analysis

CancerMIRNome (http://bioinfo.jialab-ucr.org/CancerMIRNome/, accessed on 9 September 2021) was used to analyze the expression level and prognosis of miR-1275 in The Cancer Genome Atlas (TCGA) database. Timer (cistrome.shinyapps.io/timer) was used to analyze the expression level of EIF5A2 in the TCGA database. The Kaplan–Meier (K-M) Plotter (http://kmplot.com/analysis/, accessed on 1 October 2010) was applied to analyze the effect of the EIF5A2 expression level on the survival of HCC patients in the TCGA-LIHC database. StarBase (https://starbase.sysu.edu.cn/, accessed on 1 December 2013) was adopted to predict the correlation between EIF5A2 and tumor stemness markers. Four databases (TargetScan, miRWalk, miRDB and miRPathDB) were used to predict the target genes of miR-1275. EMTome (http://www.emtome.org/, accessed on 10 December 2020) was applied to predict the downstream signaling pathways of EIF5A2, and DIANA-miRPath v3.0 (http://www.microrna.gr/miRPathv3/, accessed on 1 July 2015) was used to predict the downstream signaling pathways of miRNAs. The Kyoto Encyclopedia of Genes and Genomes (KEGG) pathway and Gene Set Variation Analysis (GSVA) enrichment analyses of miR-1275 and EIF5A2 were performed by R software version 4.1.0 (Robert Gentleman, Auckland, New Zealand).

### 2.22. Statistical Analysis

All assays were repeated at least three times, and the mean and curve graphs showed the mean and standard deviation. The data were analyzed using the Chi-squared test or Student’s *t*-test by Graphpad prism software v8.0.2 (GraphPad Software, San Diego, CA, USA). Pearson’s correlation test was applied to analyze the correlation between EIF5A2 and miR-1275. Statistical significance was defined as *p* < 0.05 (*), *p* < 0.01 (**) and *p* < 0.001 (***).

## 3. Results

### 3.1. Genistein Inhibited the Viability of HCC Cells and Upregulated miR-1275 in HCC Cells

**Figure 1:**
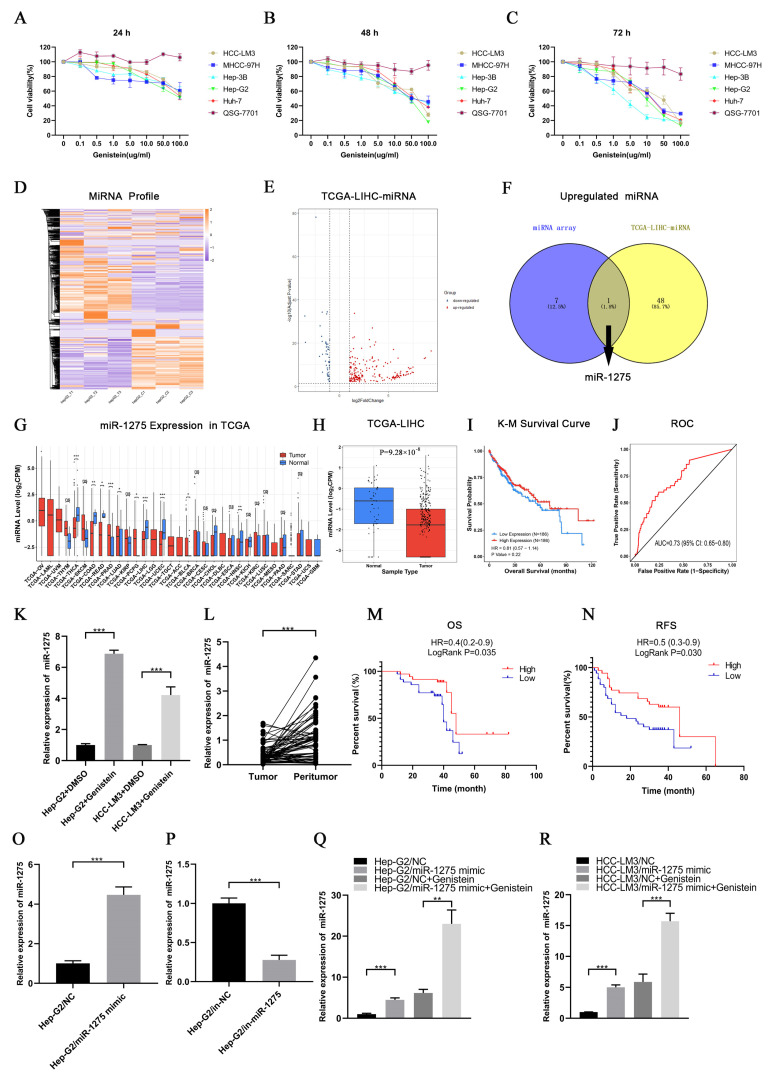
**Genistein inhibited the viability of HCC cells and upregulated miR-1275 in HCC cells.** (**A**–**C**) Cell viability of five HCC cell lines (HCC-LM3, MHCC-97H, Hep-3B, Hep-G2, Huh-7) and normal hepatocytes (QSG7701) treated with genistein (0, 0.1, 0.5, 1.0, 5.0, 10.0, 50.0 and 100.0 µg/mL) for 24 h, 48 h and 72 h. (**D**) Heatmap of miRNA expression profiles in three pairs of Hep-G2 cells treated with genistein or DMSO. (**E**) Volcano plot of differentially expressed miRNAs in the TCGA-LIHC database. Red dots represent up-regulated miRNAs and blue dots represent down-regulated miRNAs in HCC tissues (threshold, *p*-value < 0.05 and |log2FoldChange| > 1). (**F**) The Venn diagram shows the intersection of up-regulated miRNAs in the genistein treatment group and lowly expressed miRNAs in HCC tissues in the TCGA-LIHC database, and only miR-1275 meets the condition. (**G**) Pan-cancer analysis of miR-1275 expression levels in the TCGA database. (**H**) The boxplot of the expression levels of miR-1275 in HCC tissues and normal liver tissues in the TCGA-LIHC database. (**I**) K-M plot of overall survival (OS) of HCC patients with high and low expressed miR-1275 in the TCGA-LIHC database (Hazard Ratio (HR) = 0.81 and *p*-value = 0.22). (**J**) ROC plot and AUC to evaluate the accuracy of the miR-1275 expression level to predict the OS of HCC patients in the TCGA-LIHC database. (**K**) QRT-PCR analysis of the expression levels of miR-1275 in Hep-G2 and HCC-LM3 cells treated with genistein or DMSO. (**L**) QRT-PCR analysis of miR-1275 expression levels in HCC tissues and their paracancerous tissues (*n* = 70). (**M**,**N**) K-M survival analysis on OS and RFS of HCC patients with high and low expressed miR-1275, and LogRank *p*-values are shown (*n* = 70). (**O**,**P**) QRT-PCR analysis of miR-1275 expression levels in Hep-G2 cells transfected with miR-1275 mimic, mimic-NC (NC), inhibitor-NC (in-NC) and miR-1275 inhibitor (in-miR-1275). (**Q**,**R**) QRT-PCR analysis of miR-1275 expression levels in Hep-G2 and HCC-LM3 cells in four groups: NC, miR-1275 mimic, NC + genistein and miR-1275 mimic + genistein. Plotted values representing the mean ± SEM from three independent assays are provided in the images (*n* = 3). (* *p* < 0.05, ** *p* < 0.01, *** *p* < 0.001.)

First, we found that genistein had strong inhibitory effects on the viability of different HCC cell lines but had no significant effect on QSG-7701 cells. Interestingly, the inhibition was enhanced by the increase in time and the concentration of genistein. Hep-G2 cells were the most sensitive to genistein, and the IC_50_ of genistein in Hep-G2 cells at 72 h was 11.74 µg/mL. On the contrary, the IC_50_ of genistein in HCC-LM3 cells at 72 h was the highest (19.57 µg/mL) (Figure 1A–C). Therefore, we selected Hep-G2 cells treated with genistein for miRNA transcriptome sequencing, and HCC-LM3 cells were also used for subsequent experimental verification. The heatmap of sequencing results was displayed as follows (Figure 1D). The differentially expressed miRNAs between HCC samples (*n* = 372) and normal liver tissues (*n* = 50) in the TCGA-LIHC database were selected depending on the threshold (*p*-value < 0.05 and |Log2(FoldChange)| > 1) (Figure 1E). By drawing a Venn diagram, we discovered that only miR-1275 was positively regulated by genistein and was lowly expressed in HCC tissues in the TCGA-LIHC database (Figure 1F). Further pan-cancer analysis also exhibited that miR-1275 is mainly lowly expressed in a variety of tumor samples, especially in HCC tissues (Figure 1G,H). The K-M survival analysis of miR-1275 in HCC patients in the TCGA database showed that, although the LogRank test showed no significant difference (*p*-value = 0.22), the overall survival (OS) of HCC patients with a high expressed miR-1275 was higher than that of those with a low expressed miR-1275 (Figure 1I). The receiver operating characteristic (ROC) curve of the miR-1275 expression level in HCC patients in the TCGA-LIHC database was also provided, and the area under curve (AUC) was 0.73 (Figure 1J). Next, Hep-G2 and HCC-LM3 cells were treated with genistein or DMSO. As expected, the miR-1275 levels were more highly expressed in the genistein treatment group than they were in the DMSO control group (Figure 1K). However, instead, the expression level of miR-1275 changed inconspicuously in QSG-7701 cells after the treatment with genistein (Figure S1B). Similarly, in our single center, miR-1275 was shown to be more lowly expressed in HCC tissues than that in paracancerous tissues (*n* = 70) by qRT-PCR (Figure 1L). HCC patients were also grouped, and the clinical data were counted according to the expression level of miR-1275. The results were listed as a baseline table (Table 1). It showed that a low expressed miR-1275 was associated with HCC proliferation (large tumor size). K-M survival analysis exhibited that the OS and relapse-free survival (RFS) of HCC patients with highly expressed miR-1275 were higher than those of those with a low expression level (*p*-value < 0.05) (Figure 1M,N). In order to explore the effects of miR-1275 on Hep-G2 and HCC-LM3 cells, we overexpressed and knocked down miR-1275 separately, and the efficiency of transfection in Hep-G2 cells was measured by qRT-PCR (Figure 1O,P). Moreover, the expression level of miR-1275 in Hep-G2 and HCC-LM3 cells co-treated with miR-1275 mimic and genistein was significantly higher than that in other groups (Figure 1Q,R). Thus, genistein can inhibit the viability of HCC cells and raise the expression level of miR-1275 in HCC cells.

### 3.2. Genistein Inhibited the EMT and Stemness of HCC Cells by Upregulating miR-1275 In Vitro

**Figure 2:**
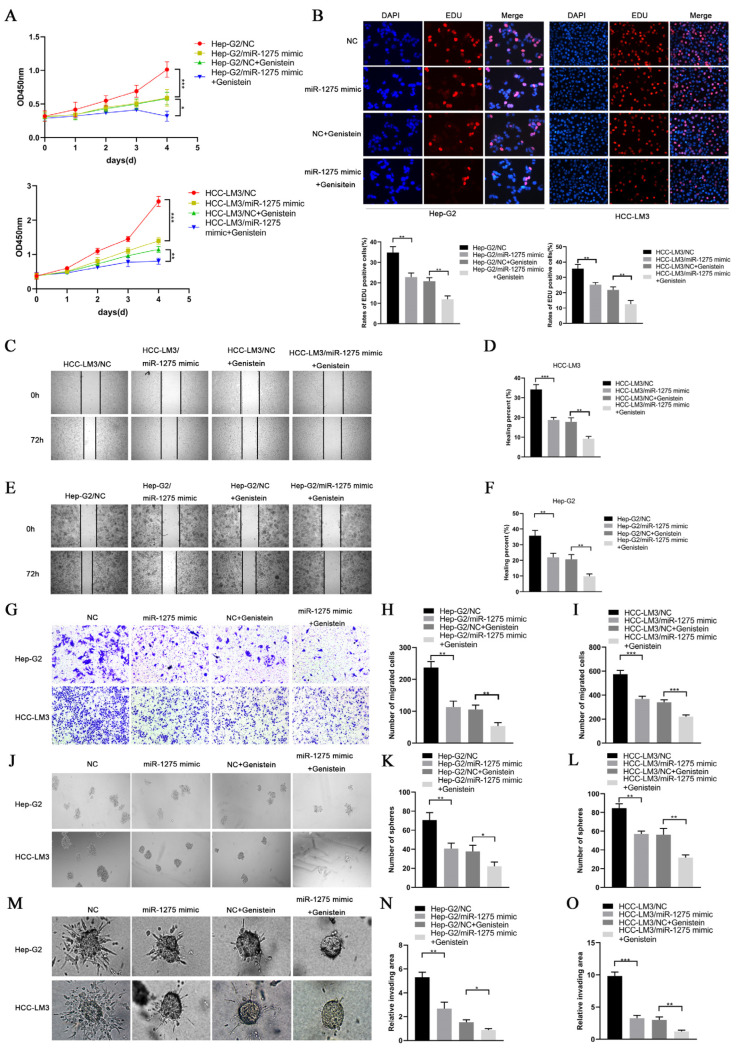
**MiR-1275 upregulated by genistein inhibited the progression of HCC cells in vitro.** (**A**) CCK-8 assays for evaluating the proliferation of Hep-G2 and HCC-LM3 cells in four groups: NC, miR-1275 mimic, NC + genistein and miR-1275 mimic + genistein. (**B**) EDU assays for assessing the proliferation of Hep-G2 and HCC-LM3 cells in the above four groups. DAPI (blue) represents all cells; EDU (red) represents proliferative cells. (**C**–**F**) The scratch-healing tests for assessing the migration of Hep-G2 and HCC-LM3 cells in the above four groups. The vertical lines on both sides in the images represent the boundaries of the scratch. (**G**–**I**) Transwell migration assays for evaluating the migration of Hep-G2 and HCC-LM3 cells in the above four groups. (**J**–**L**) The spheroid formation tests for assessing the stemness of Hep-G2 and HCC-LM3 cells in the above four groups. (**M**–**O**) 3D spheroid invasion assays for evaluating the invasion of Hep-G2 and HCC-LM3 cells in the above four groups. Plotted values representing the mean ± SEM from three independent assays are provided in the images (*n* = 3). (* *p* < 0.05, ** *p* < 0.01, *** *p* < 0.001.)

**Figure 3:**
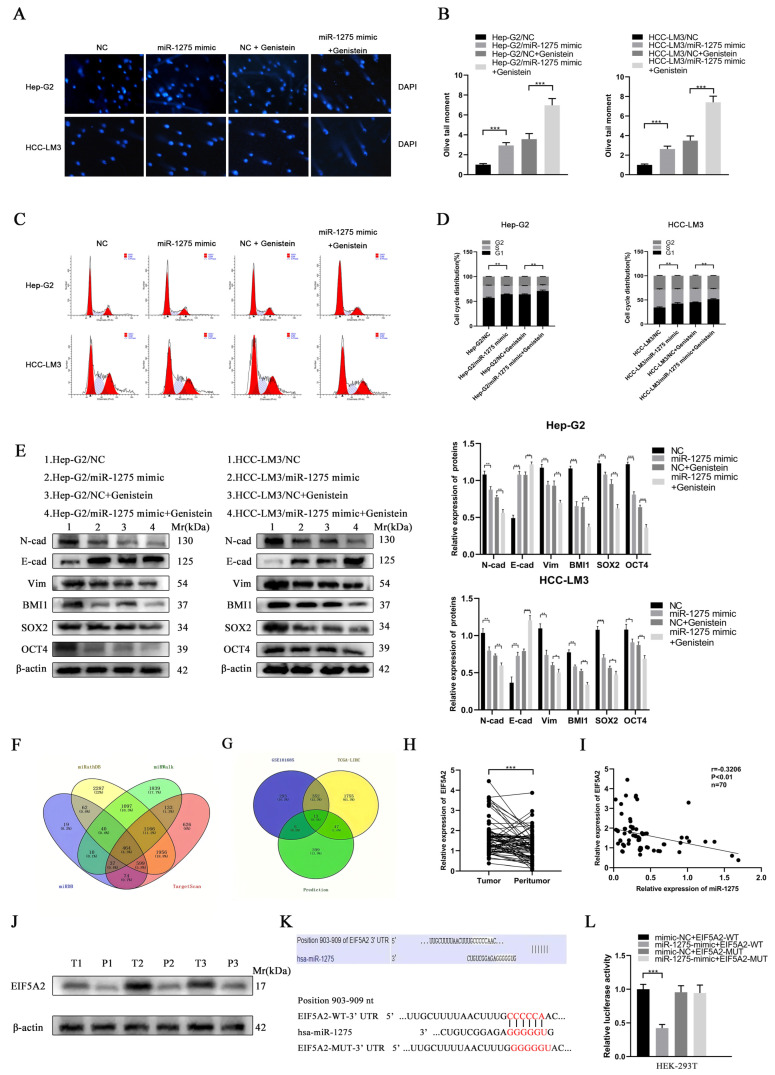
**Genistein upregulated miR-1275 to suppress the EMT and stemness of HCC cells through the targeted inhibition of EIF5A2.** (**A**,**B**) Comet Assays for evaluating the DNA damage and apoptosis of Hep-G2 and HCC-LM3 cells in four groups: NC, miR-1275 mimic, NC + genistein and miR-1275 mimic + genistein. (**C**,**D**) The flow cytometry analysis on cell cycles of Hep-G2 and HCC-LM3 cells in the above four groups. (**E**) WB analysis on the expression levels of EMT protein markers (E-cadherin, N-cadherin, Vimentin) and stemness protein markers (BMI1, SOX2, OCT4) in Hep-G2 and HCC-LM3 cells in the above four groups. (**F**) Venn diagram shows the intersection of four databases (TargetScan, miRWalk, miRDB and miRPathDB) that predicted the target genes of miR-1275. (**G**) Venn diagram shows the intersection of the predicted target genes of miR-1275 in the above four databases and the up-regulated genes in HCC tissues in the TCGA-LIHC and GSE101685 databases. (**H**) QRT-PCR analysis of the expression levels of miR-1275 in HCC tissues and their paracancerous tissues (*n* = 70). (**I**) Pearson’s correlation analysis of the expression levels between miR-1275 and EIF5A2 in HCC tissues. Pearson’s correlation coefficient (r) and *p*-value are shown (*n* = 70). *p*-value is from Pearson’s test. (**J**) WB analysis of EIF5A2 expression levels in HCC tissues and their paracancerous tissues (*n* = 3). (**K**) Original and mutated 3′UTR sites of EIF5A2 potential to bind miR-1275 predicted by the TargetScan database. (**L**) Luciferase activity was analyzed in HEK293T cells co-transfected with WT EIF5A2-3′-UTR or MUT EIF5A2-3′-UTR and miR-1275 mimic or negative control oligoribonucleotides. Plotted values representing the mean ± SEM from three independent assays are provided in the images (*n* = 3). (* *p* < 0.05, ** *p* < 0.01, *** *p* < 0.001.)

EMT and stemness promote the progression and metastasis of HCC cells. In order to determine the effects of genistein and miR-1275 on the EMT and stemness of Hep-G2 and HCC-LM3 cells, we performed a series of in vitro functional experiments. In the proliferative experiment, the CCK-8 assay and EDU test were performed and indicated that the overexpression of miR-1275 or genistein treatment can restrain the proliferation of Hep-G2 and HCC-LM3 cells. Moreover, this inhibitory effect was further enhanced when combined with miR-1275 mimic and genistein (Figure 2A,B). Similarly, the same result was obtained from the motility experiments. The scratch-healing test and transwell migration assay were conducted and exhibited that the combination of miR-1275 mimic and genistein can further suppress the migration of Hep-G2 and HCC-LM3 cells (Figure 2C–I). Then, the spheroid formation test was applied to assess the stemness of Hep-G2 and HCC-LM3 cells, and it proved that miR-1275 mimic and genistein can synergistically attenuate the stemness of Hep-G2 and HCC-LM3 cells (Figure 2J–L). In order to better simulate the invasion of tumor cells, we performed a 3D spheroid invasion assay and discovered that miR-1275 mimic and genistein can also cooperate to inhibit the invasion of Hep-G2 and HCC-LM3 cells (Figure 2M–O). In addition, the Comet Assay was conducted to prove that the synergistic effect of miR-1275 mimic and genistein significantly aggravated DNA damage in Hep-G2 and HCC-LM3 cells and accelerated their apoptosis (Figure 3A,B).

The flow cytometry analysis on Hep-G2 and HCC-LM3 cells also confirmed that miR-1275 mimic and genistein cooperatively decreased the proportion of S phase and increased the proportion of G0/G1 phase to further limit the proliferation of Hep-G2 and HCC-LM3 cells (Figure 3C,D). Finally, the expression levels of EMT and stemness markers in Hep-G2 and HCC-LM3 cells in four groups were measured by WB analysis. The results exhibited that, except for the increased expression of E-cadherin, the EMT markers (N-cadherin and Vimentin) and the stemness markers (SOX2, BMI1 and OCT4) in Hep-G2 and HCC-LM3 cells were declined by genistein and miR-1275 mimic (Figure 3E). Combined with previous qRT-PCR analysis results, it was clear that, under the synergistic effect of miR-1275 mimic and genistein, miR-1275 was significantly raised and further weakened the EMT and stemness of Hep-G2 and HCC-LM3 cells. Therefore, these in vitro functional experiments proved that miR-1275 upregulated by genistein can suppress HCC progression by inhibiting the EMT and stemness of HCC cells.

### 3.3. EIF5A2 Was the Target Gene of miR-1275 in HCC Cells

To explore the target gene of miR-1275, we obtained the intersection of four target gene prediction databases (TargetScan, miRWalk, miRDB and miRPathDB) of miR-1275 online, and the Venn diagram was also mapped (Figure 3F). However, due to the large number of predicted genes, we selected the genes that were highly expressed (threshold, *p*-value < 0.05 and log2FoldChange > 1) in HCC tissues in the TCGA-LIHC database and the Gene Expression Omnibus (GEO) database (GSE101685). There were 13 potential target genes of miR-1275 that qualified (Figure 3G). After further screening, we found that only EIF5A2 met the conditions. To confirm our conjecture, the expression levels of EIF5A2 in 70 pairs of HCC and matched adjacent tissues were analyzed by qRT-PCR, and it indicated that EIF5A2 was more highly expressed in HCC tissues than it was in peritumor tissues (Figure 3H). Meanwhile, Pearson’s correlation analysis showed that miR-1275 and EIF5A2 were negatively correlated in HCC tissues (*n* = 70). The Pearson’s correlation coefficient (r) and *p*-value (P) were also shown (Figure 3I). The protein levels of EIF5A2 were also found to be highly expressed in HCC tissues compared with adjacent tissues (*n* = 3) (Figure 3J). To determine the binding site of the 3′UTR region of EIF5A2 and miR-1275, the predicted binding site acquired from the TargetScan database and the mutant sequence of 3′UTR of EIF5A2 were listed (Figure 3K). A subsequent dual luciferase reporter test also confirmed that the luciferase activity of HEK293T cells co-transfected with miR-1275 mimic and EIF5A2-WT-3′UTR was lower than that in other groups (Figure 3L). Therefore, we proved that EIF5A2 was highly expressed in HCC tissues, and it was the target gene of miR-1275 in HCC cells.

### 3.4. MiR-1275 Upregulated by Genistein Suppressed the Progression and Metastasis of HCC by Inhibiting EIF5A2 In Vivo

**Figure 4:**
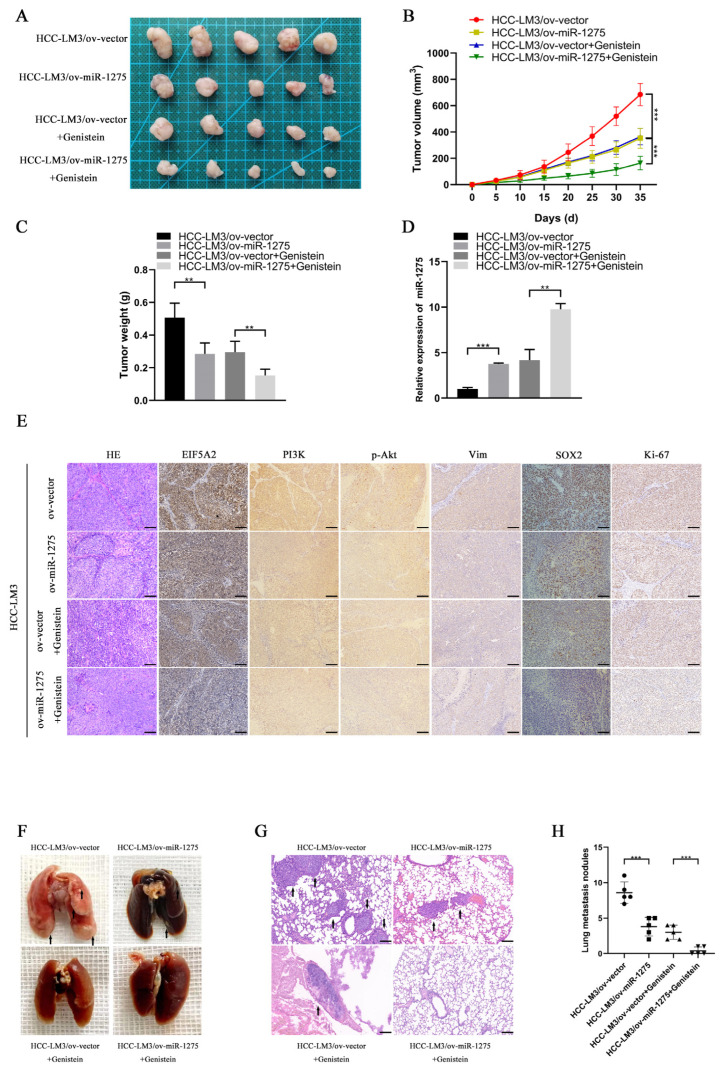
**MiR-1275 upregulated by genistein inhibited the progression and metastasis of HCC by attenuating the EMT and stemness of HCC in vivo.** (**A**) The photograph of xenografts obtained from the nude mice injected by HCC-LM3 cells in four groups: ov-vector, ov-miR-1275, ov-vector + genistein and ov-miR-1275 + genistein (*n* = 5). (**B**,**C**) Growth curve of tumor volumes and the weights of the xenograft tumors in the above four groups. (**D**) QRT-PCR analysis of miR-1275 expression levels in the xenograft tumors in the above four groups. (**E**) H&E staining and immunohistochemical staining analysis of the expression levels of EIF5A2, PI3K, p-Akt, Vimentin, SOX2 and Ki-67 in xenograft tumors in the above four groups. Scale bar represents 100 µm. (**F**,**G**) The photograph and H&E staining images of lung metastasis obtained from the nude mice injected by HCC-LM3 cells in the above four groups (*n* = 5), and the lung metastatic nodules are indicated by arrows. Scale bar represents 100 µm. (**H**) The number of lung metastatic nodules in the above four groups (*n* = 5). Plotted values representing the mean ± SEM are provided in the images (*n* = 5). (** *p* < 0.01, *** *p* < 0.001.)

To explore whether miR-1275 and genistein can exert the same effect in in vivo experiments, we constructed a xenograft tumor model and lung metastasis model of HCC-LM3 cells in nude mice. The transfection efficiency of ov-miR-1275 lentivirus in HCC-LM3 cells was shown, as follows (Figure S1C). The Xenograft HCC model showed that the volumes and weights of tumors in the genistein treatment group and the miR-1275 overexpression group (ov-miR-1275) were significantly smaller than those in the control group (ov-vector). Furthermore, the volumes and weights of tumors in the ov-miR-1275 + genistein group were the smallest (Figure 4A–C). The subsequent qRT-PCR analysis of miR-1275 expression levels in tumors proved that the miR-1275 in tumors of the ov-miR-1275 + genistein group was expressed more highly than that in other groups (Figure 4D). These results were consistent with previous in vitro assays. On the contrary, the immunohistochemical analysis of xenografts exhibited that the expression level of EIF5A2 in tumors of the ov-miR-1275 + genistein group was lower than that in other groups. In the same way, the expression levels of proteins (PI3K, p-Akt, Vimentin, SOX2 and Ki-67) in the ov-miR-1275 + genistein group were also the lowest (Figure 4E). In addition, genistein treatment and the overexpression of miR-1275 decreased the number of metastatic nodules in lung tissues, and the inhibition was the strongest in the ov-miR-1275 + genistein group (Figure 4F–H). These in vivo experiments proved that miR-1275, upregulated by genistein, can suppress the progression and metastasis of HCC by inhibiting EIF5A2.

### 3.5. MiR-1275 Upregulated by Genistein Attenuated the EMT and Stemness of HCC Cells by Inhibiting the EIF5A2/PI3K/Akt Signaling Pathway

**Figure 5:**
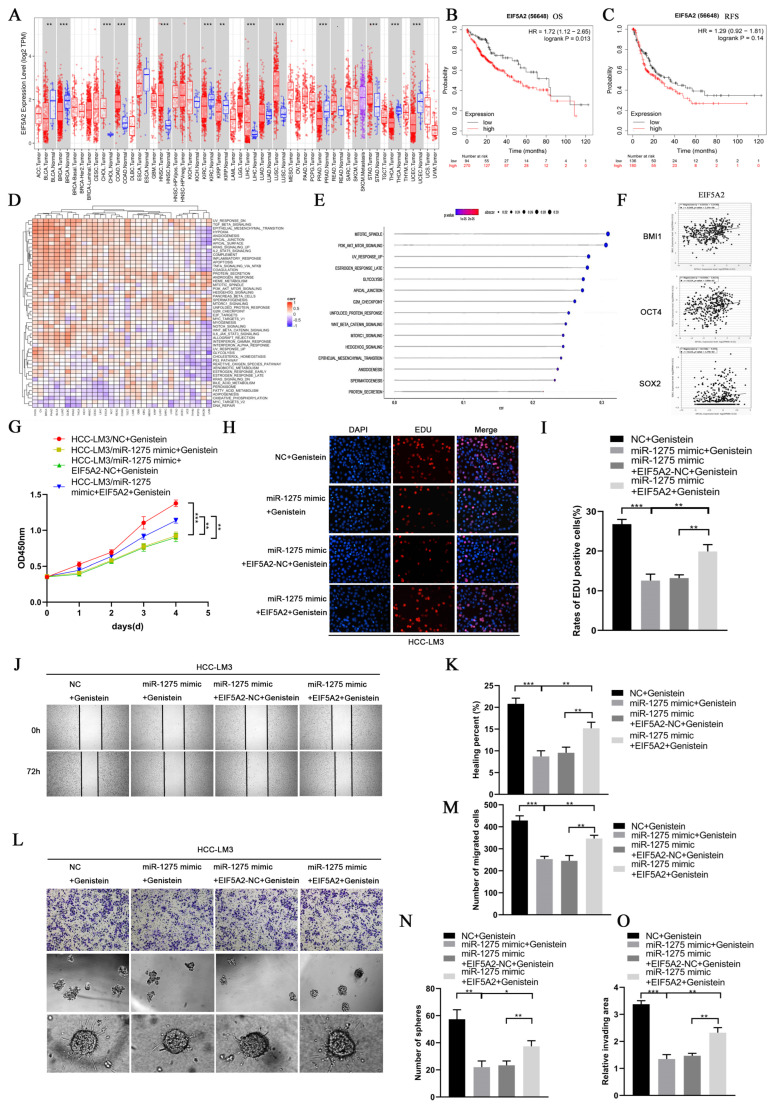
**The inhibitory effect of miR1275 upregulated by genistein on the EMT and stemness of HCC can be reversed by EIF5A2 in vitro.** (**A**) Pan-cancer analysis of EIF5A2 expression levels in the TCGA database. (**B**,**C**) K-M survival analysis of the OS and RFS of HCC patients with high or low expressed EIF5A2 in the TCGA-LIHC database. (**D**) The heatmap of the GSVA enrichment analysis of EIF5A2 in the TCGA database. (**E**) The GSVA enrichment analysis of EIF5A2 expression levels in HCC tissues in the TCGA-LIHC database (threshold, *p*-value < 0.05). (**F**) Pearson’s correlation analysis of the expression levels between EIF5A2 and stemness markers (BMI1, OCT4 and SOX2) in HCC tissues in the TCGA-LIHC database. Pearson’s correlation coefficient (r) and *p*-value are shown (*p* < 0.05). *p*-value is from Pearson’s test. (**G**) CCK-8 assays for assessing the proliferation of HCC-LM3 cells in four groups: NC + genistein, miR-1275 mimic + genistein, miR-1275 mimic + EIF5A2-NC+ genistein and miR-1275 mimic+ EIF5A2 + genistein. (**H**,**I**) The EDU assays for evaluating the proliferation of HCC-LM3 cells in the above four groups. DAPI (blue) represents all cells; EDU (red) represents proliferative cells. (**J**,**K**) The scratch-healing tests for assessing the migration of HCC-LM3 cells in the above four groups. The vertical lines on both sides in the images represent the boundaries of the scratch. (**L**–**O**) Transwell migration, spheroid formation and 3D spheroid invasion assays for assessing the migration, stemness and invasion of HCC-LM3 cells in the above four groups. Plotted values representing the mean ± SEM from three independent assays are provided in the images (*n* = 3). (* *p* < 0.05, ** *p* < 0.01, *** *p* < 0.001.)

EIF5A2 is a key gene known to regulate the EMT and stemness of various tumors [21,22]. In order to explore the common downstream signaling pathway regulated by miR-1275 and EIF5A2 in HCC cells, we performed bioinformatics analysis on EIF5A2 and miR-1275. First, based on the previous transcriptome sequencing results, we performed KEGG pathway enrichment analysis (threshold, *p*-value < 0.05) on the top 18 miRNAs with obvious differential expression levels. The signaling regulation network was constructed by DIANA-miRPath v3.0 database and R software. We found that miRNAs regulated by genistein mainly dominated the downstream PI3K-Akt signaling pathway (Figure S1A). Then, a pan-cancer analysis of EIF5A2 in the TCGA database indicated that EIF5A2 was highly expressed in various tumor tissues, including HCC (Figure 5A). According to the K-M survival analysis, the OS and RFS of HCC patients with highly expressed EIF5A2 in the TCGA-LIHC database were lower than those of those with lowly expressed EIF5A2 (Figure 5B,C). Meanwhile, the GSVA analysis of EIF5A2 in the TCGA database was conducted, and the heatmap was mapped by the EMTome database (Figure 5D). The GSVA analysis of EIF5A2 and the screened related signaling pathways (threshold, *p*-value < 0.05) in the TCGA-LIHC database were also shown in the chart. Interestingly, EIF5A2 was positively correlated with the PI3K-Akt signaling pathway (Figure 5E). Moreover, EIF5A2 was found to be positively correlated with EMT and the stemness markers of HCC in the TCGA-LIHC database (Figure S1D and Figure 5F). Therefore, we suspected that miR-1275 may attenuate the EMT and stemness of HCC cells by inhibiting the EIF5A2/PI3K/Akt signaling pathway. Finally, immunohistochemical staining and WB analyses confirmed that the expression levels of EIF5A2, PI3K and p-Akt declined evidently in Hep-G2 and HCC-LM3 cells treated with genistein miR-1275 mimic, but Akt changed inapparently (Figure S1E,F and Figure 4E). Thus, it can be concluded that miR-1275 upregulated by genistein can suppress the EMT and stemness of HCC cells by inhibiting the EIF5A2/PI3K/Akt signaling pathway.

### 3.6. The Inhibitory Effect of miR-1275 on the EMT and Stemness of HCC Can Be Reversed by EIF5A2

**Figure 6:**
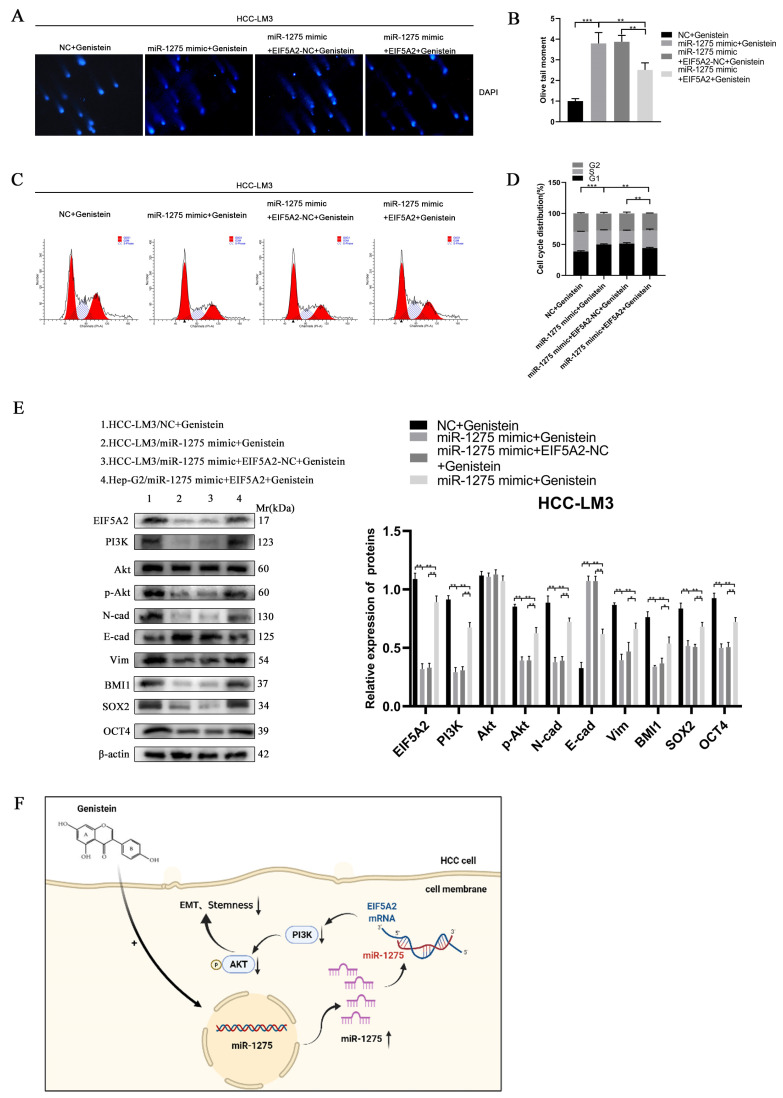
**The inhibitory effect of miR-1275 upregulated by genistein on the EMT and stemness of HCC can be reversed by the activation of the EIF5A2/PI3K/Akt signaling pathway.** (**A**,**B**) Comet Assay for evaluating the DNA damage and apoptosis of HCC-LM3 cells in four groups: NC + genistein, miR-1275 mimic + genistein, miR-1275 mimic + EIF5A2-NC + genistein and miR-1275 mimic + EIF5A2 + genistein. (**C**,**D**) The flow cytometry analysis on cell cycles of HCC-LM3 cells in the above four groups. (**E**) WB analysis on the expression levels of EIF5A2, PI3K, Akt, p-Akt, EMT markers (E-cadherin, N-cadherin, Vimentin) and stemness markers (BMI1, SOX2, OCT4) in HCC-LM3 cells in the above four groups. (**F**) A proposed model illustrating the role of miR-1275 upregulated by genistein in inhibiting the EMT and stemness of HCC cells. Plotted values representing the mean ± SEM from three independent assays are provided in the images (*n* = 3). (* *p* < 0.05, ** *p* < 0.01, *** *p* < 0.001.)

In order to determine whether EIF5A2 can enhance the EMT and stemness of HCC, HCC-LM3 cells were co-transfected with miR-1275 mimic and EIF5A2 overexpression or EIF5A2-NC plasmid. At the same time, the HCC-LM3 cells in the four groups were all treated with genistein. The CCK-8 and EDU tests suggested that the proliferation ability of HCC-LM3 cells transfected with EIF5A2 overexpression plasmid was stronger than that in the EIF5A2-NC group and miR-1275 mimic group (Figure 5G–I). The scratch-healing and transwell migration assays also exhibited that the inhibitory effect of miR-1275 mimic on the migration of HCC-LM3 cells was partially weakened by EIF5A2 overexpression plasmid but not by EIF5A2-NC plasmid (Figure 5J–M). Moreover, in the spheroid formation and 3D spheroid invasion assays, the stemness and invasion of HCC-LM3 cells in the miR-1275 mimic+ EIF5A2 group were enhanced to some extent compared with that in the miR-1275 mimic + EIF5A2-NC group or miR-1275 mimic group (Figure 5L,N,O). The Comet Assay indicated that the DNA damage of HCC-LM3 cells in the miR-1275 mimic + EIF5A2 group was alleviated compared with that in the miR-1275 mimic + EIF5A2-NC or miR-1275 mimic groups (Figure 6A,B). The flow cytometry analysis found that, compared with the miR-1275 mimic + EIF5A2-NC and miR-1275 mimic groups, the proportion of the S phase was raised and the proportion of the G0/G1 phase was reduced in the miR-1275 mimic+ EIF5A2 group (Figure 6C,D). Finally, the WB analysis found that the expression level of EIF5A2 in the miR-1275 mimic+ EIF5A2 group was improved compared with that in the miR-1275 mimic or miR-1275 mimic + EIF5A2-NC group, but it was still lower than that in the NC group. In addition, compared with other groups, the expression levels of PI3K, p-Akt, EMT markers (N-cadherin and Vimentin) and stemness markers (SOX2, BMI1 and OCT4) in the miR-1275 mimic+ EIF5A2 group had the same trend as those in the EIF5A2 group. However, the expression level of the EMT marker (E-cadherin) in the miR-1275 mimic+ EIF5A2 group was the opposite, and there was no significant difference in the expression levels of Akt among the four groups (Figure 6E). From this, we drew a conclusion that the inhibitory effect of miR-1275 on the EMT and stemness of HCC can be reversed by the activation of the EIF5A2/PI3K/Akt signaling pathway. The pattern diagram of interpreting the miR-1275/EIF5A2/PI3K/Akt axis in HCC-LM3 cells was exhibited (Figure 6F). Therefore, it was clear that genistein can positively regulate miR-1275 to suppress the EMT and stemness of HCC cells by inhibiting the EIF5A2/PI3K/Akt signaling pathway in vivo and vitro.

## 4. Discussion

Genistein (C_15_H_10_O_5_, Mr = 270.24) accounts for 60% of soybean isoflavones (SIF) in soybeans [23]. In previous studies, genistein was mainly focused on for its role in inhibiting tumor growth or inducing apoptosis. Similarly, it has been well demonstrated that genistein has the ability to inhibit HCC progression and induce HCC apoptosis [13,24]. At the initial stage of our study, we determined that the inhibitory effects on HCC viability were dependent both on the time and the doses of genistein treatment, and the results were consistent with other previous studies [8,9,13]. On these grounds, we raised the question of through which way genistein can regulate HCC progression and metastasis. Then, we paid attention to the EMT and stemness of HCC. On the one hand, the occurrence of EMT leads to a decrease in cancer cells adhesion and an enhancement of their invasion and metastasis. On the other hand, the enhancement of stemness makes cancer cells possess powerful self-renewal and multidirectional differentiation abilities [3,25]. Briefly speaking, EMT and stemness are indispensable in promoting the progression and metastasis of cancers. Next, we further speculated whether genistein can exert an anti-tumor effect by abating the stemness and EMT of HCC cells. In addition, it has also been widely reported that miRNAs play an important role in dominating the EMT and stemness of HCC [2,26]. For example, miR-192-5p and miR-568 were found to regulate the stemness behavior to affect HCC progression [27,28]. In our study, we found for the first time that miR-1275 can be positively regulated by genistein in HCC cells through transcriptome sequencing and series experiments. Before our study, the miRNA array analysis of HCC tissues performed by Wen Wang et al. [29] and other studies also supported that miR-1275 is involved in inhibiting the progression and metastasis of HCC [30]. As indicated earlier, miR-1275 is essential for genistein to inhibit HCC progression and metastasis. In addition, the EMT and stemness of HCC cells were indeed weakened by miR-1275 upregulation according to the results of immunohistochemistry and WB. It proved that miR-1275 abated the EMT and stemness to further suppress the progression and metastasis of HCC.

It is a priority to figure out the key gene and common signaling pathway between EMT and stemness in controlling HCC progression and metastasis. Through prediction by databases and the dual luciferase reporter test, EIF5A2 was found to have potential to control the stemness and EMT of HCC. EIF5A2 (eukaryotic initiation factor 5A2) is located at 3q26 on human chromosomes [21]. EIF5A2 can act as a transcription factor to drive the occurrence of multiple cancers and induce poor prognosis [21]. EIF5A2 has the effect of enhancing the EMT and stemness of tumors, which promotes the progression, recurrence and metastasis of cancers [31,32]. For example, it was reported that EIF5A2 was involved in maintaining the existence of cancer stem cells in HCC cells through the c-Myc pathway [32]. In addition, Zhiyuan Zhang et al. [31] confirmed that EIF5A2 can improve the EMT to promote the metastasis of colorectal cancer. Overall, EIF5A2 is the key gene that simultaneously regulates the interaction between EMT and stemness. Meanwhile, there are many common signaling pathways between EMT and stemness, such as PI3K/Akt, Wnt/β-catenin and TGF-β [33,34,35]. Because of this, through KEGG and GSVA enrichment analyses, we realized that EIF5A2/PI3k/Akt was the key signaling pathway regulating HCC EMT and stemness. PI3K (phosphatidylinositol kinase) is a dimer composed of regulatory subunit p85 and catalytic subunit p110. The upregulation of PI3K can change the structure of Akt and activate it by phosphorylation, thus regulating cell proliferation, differentiation, apoptosis, migration and other phenotypes [34,36]. The PI3K/Akt pathway has proven that it can be activated by the degradation of P85a or RALYL to enhance the stemness and promote the EMT of HCC [3,36]. The knockdown of EIF5A2 can restrain the PI3K/Akt signaling pathway to prevent the growth of bladder cancer [37]. Subsequent WB and immunohistochemical analysis also exhibited that EIF5A2/PI3K/Akt attenuated by miR-1275 and genistein suppressed the EMT and stemness of HCC. This inhibitory effect induced the retardation of HCC progression and metastasis. Moreover, the DNA damage of HCC cells was aggravated, and cell cycles were also blocked in the GO/G1 phase. In view of this, miR-1275 upregulated by genistein can indeed inhibit the EIF5A2/PI3K/Akt pathway to suppress the EMT and stemness of HCC.

Despite all this, there were still some deficiencies in this study. First, the concentrations of genistein used in our study were consistent with the recommended concentration range (2.7–27.0 µg/mL) [8,9,13]. However, several scholars pointed out that different doses of genistein have different effects on tumors, and the specific drug concentration of genistein is still controversial [38,39,40]. In the follow-up study, we will further explore the anti-tumor mechanisms of genistein at different concentrations. Second, the tumor microenvironment (TME) is also very important to the crosstalk between EMT and stemness, and we will focus on the effects of genistein on the communication between HCC cells and other cells in TME.

In summary, genistein has powerful anti-HCC effects and can play a variety of roles to inhibit the progression and metastasis of HCC. In our study, we confirmed that miR-1275 upregulated by genistein can attenuate HCC progression and metastasis by suppressing the EIF5A2/PI3K/Akt signaling pathway to inhibit the EMT and stemness of HCC cells. It is clear that genistein can control the expression levels of miRNAs to restrain HCC. However, the dose and duration of genistein are still controversial, and it will take more time to be verified. Our research provides a new direction and theoretical basis for seeking new adjuvant drugs for HCC, and miR-1275 may become a new therapeutic target of HCC.

## 5. Conclusions

In summary, our study indicated that miR-1275 upregulated by genistein can attenuate the EMT and stemness of HCC by inhibiting the EIF5A2/PI3K/Akt signaling pathway and thus restrict HCC progression and metastasis.

## Figures and Tables

**Table 1:** Clinical characteristics of HCC patients (*n* = 70) grouped according to the expression level of miR-1275.

Clinicopathological	Number	Low (*n* = 35)	High (*n* = 35)	*p* Value
Parameters		miR-1275	miR-1275	
Age (years)	70			1.000
≤60	52	26	26	
>60	18	9	9	
Gender	70			0.480
Female	10	4	6	
Male	60	31	29	
Liver cirrhosis	70			0.255
Yes	54	25	29	
No	16	10	6	
HBsAg	70			0.255
Positive	54	25	29	
Negative	16	10	6	
Virus titer (copies/mL)	70			0.434
≤100	49	23	26	
>100	21	12	9	
AFP (ng/mL)	70			0.334
≤20	30	17	13	
>20	40	18	22	
DCP (ng/mL)	70			0.803
≤40	45	22	23	
>40	25	13	12	
Tumor size (cm)	70			0.008 *
≤5	37	13	24	
>5	33	22	11	
Tumor multiplicity	70			0.495
Single	60	29	31	
Multiple	10	6	4	
Microvascular invasion	70			0.597
Yes	20	11	9	
No	50	24	26	
Edmondson grade	70			0.584
I–II	52	27	25	
III–IV	18	8	10	
TNM stage	70			0.075
I–II	47	20	27	
III–IV	23	15	8	

* Indicates *p* < 0.05; AFP: a-fetoprotein; DCP: des-γ-carboxy prothrombin.

## Data Availability

Data are contained within the article and Supplementary Materials.

## Supplementary Materials

The following supporting information can be downloaded at: https://www.mdpi.com/article/10.3390/biology11101383/s1, Figure S1. MiR-1275 upregulated by genistein inhibited the EMT and stemness of HCC by attenuating the EIF5A2/PI3K/Akt signaling pathway. (A) The KEGG pathway enrichment analysis and the regulatory network of the top 18 differentially expressed miRNAs in Hep-G2 cells treated with genistein compared with those in the control group. (*n* = 3) (threshold, *p*-value < 0.05). (B) QRT-PCR analysis of expression levels of miR-1275 in QSG-7701 cells treated with genistein and DMSO. (C) QRT-PCR analysis of expression levels of miR-1275 in HCC-LM3 cells transfected with ov-miR-1275 lentivirus and ov-vector. (D) The Spearman’s correlation analysis between EIF5A2 expression level and EMT, tumor proliferation, DNA replication and DNA repair in HCC tissues in the TCGA-LIHC database. Spearman’s correlation coefficient (r) and *p*-value are shown. *p*-value is from Spearman’s test. (E,F) WB analysis of expression levels of EIF5A2, PI3K, Akt and p-Akt in Hep-G2 and HCC-LM3 cells in four groups: NC, miR-1275 mimic, NC + genistein and miR-1275 mimic + genistein. Plotted values representing the mean ± SEM from three independent assays are provided in the images (*n* = 3). (* *p*< 0.05, ** *p* < 0.01, *** *p* < 0.001); Table S1: Primer sequence designed for qRT-PCR.
