# Supplementary material for: Genistein Restricts the Epithelial Mesenchymal Transformation (EMT) and Stemness of Hepatocellular Carcinoma via Upregulating miR-1275 to Inhibit the EIF5A2/PI3K/Akt Pathway
Source: Biology (Basel). 2022 Sep 22;11(10):1383. doi: 10.3390/biology11101383 (PMC9598820; doi:10.3390/biology11101383)
Supplement: Supplementary file 1 [file biology-11-01383-s001.zip › Table S1.pdf]

Table S1.

Primer sequence designed for qRT-PCR

Name	Forward	Reverse
miR-1275	5’-CTCTGTGAGAAAGGGTGTGG-3’	5’-TCTGCCTTGGGAAAATAAG-3’
U6	5’-GCTTCGGCAGCACATATACTAAA-3’	5’-GCTTCACGAATTTGCGTGTCAT-3’
EIF5A2	5′-GCAGACGAAAUUGAUUUCATT-3′	5′-UGAAAUCAAUUUCGUCUGCTT-3′
GAPDH	5’-ATGGAAATCCCATCACCATCT-3’	5’-GGTTGAGCACAGGGTACTTTATT-3’
