# Supplementary figures and images for: Genistein Restricts the Epithelial Mesenchymal Transformation (EMT) and Stemness of Hepatocellular Carcinoma via Upregulating miR-1275 to Inhibit the EIF5A2/PI3K/Akt Pathway
Source: Biology (Basel). 2022 Sep 22;11(10):1383. doi: 10.3390/biology11101383 (PMC9598820; doi:10.3390/biology11101383)

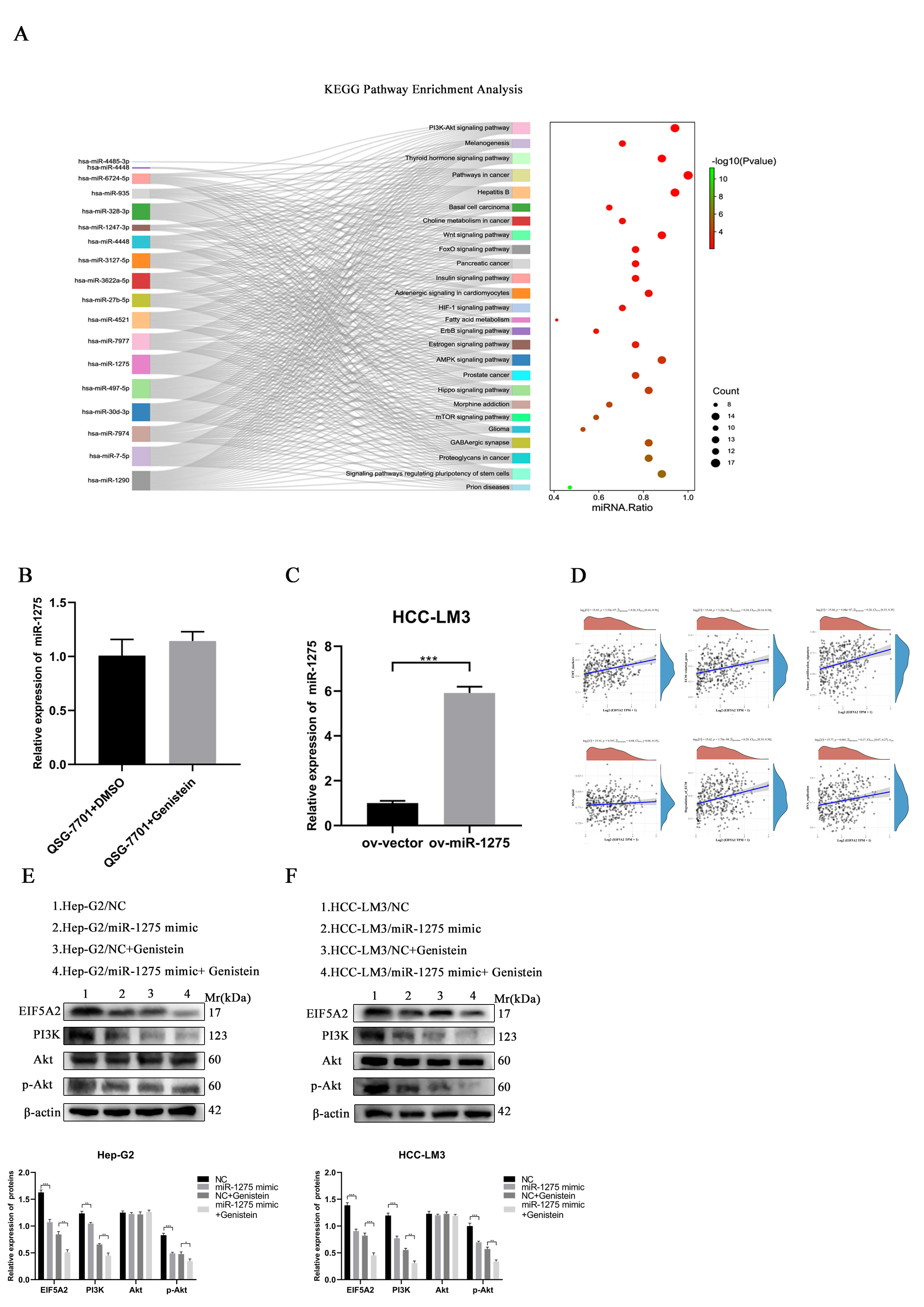

Supplement: Supplementary file 1 [file biology-11-01383-s001.zip › Figure S1.jpg]
